# Heatwave‐induced functional shifts in zooplankton communities result in weaker top‐down control on phytoplankton

**DOI:** 10.1002/ece3.70096

**Published:** 2024-08-06

**Authors:** Thu‐Hương Huỳnh, Zsófia Horváth, Károly Pálffy, Vivien Kardos, Beáta Szabó, Péter Dobosy, Csaba F. Vad

**Affiliations:** ^1^ Institute of Aquatic Ecology HUN‐REN Centre for Ecological Research Budapest Hungary; ^2^ Doctoral School of Biology, Institute of Biology Eötvös Loránd University Budapest Hungary; ^3^ National Multidisciplinary Laboratory for Climate Change HUN‐REN Centre for Ecological Research Budapest Hungary

**Keywords:** climate change, functional traits, heatwave, microzooplankton, species interactions, top‐down control, warming

## Abstract

Freshwater ecosystems are increasingly affected by rising annual mean temperatures and heatwaves. While heatwaves are expected to have more immediate effects than mean temperature increases on local communities, comparative experimental studies are largely lacking. We conducted a 1‐month mesocosm experiment to test the effect of different warming treatments, constantly raised temperatures (+3°C) and recurring heatwaves (+6°C), on plankton communities. We specifically tested how shifts in zooplankton trait composition and functional groups are reflected in ecosystem function (top‐down control on primary producers). We found that heatwaves had a stronger and more immediate effect on zooplankton trait composition (specifically on body length and body mass) and functional groups. Heatwaves led to the decrease of small‐bodied grazers (i.e., Rotifera) and the dominance of larger omnivorous Copepoda, and these shifts resulted in weaker top‐down control, leading to elevated phytoplankton biomass. Altogether, our results highlight the importance of the indirect effects of heatwaves via inducing shifts in zooplankton functional groups and trait composition, which may lead to algal blooms.

## Graphical abstract

In an outdoor mesocosm experiment, we tested the effect of constantly raised temperatures (+3°C) and recurring heatwaves (+6°C) on plankton communities for 1 month. Our work provides experimental evidence for changes in top‐down control on algal biomass in response to heatwaves, modulated through changing community size distribution and functional composition of zooplankton.

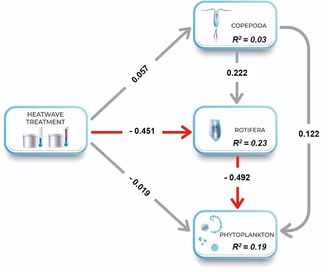

## 1 INTRODUCTION

Heatwaves, broadly defined as periods of excessively hot temperature conditions relative to the average values of a given region and season, are expected to occur with increasing intensity, duration, and frequency under climate change (IPCC, 2023; Marx et al., 2021; Woolway et al., 2021). Compared to rising annual mean temperature, heatwaves might exert a stronger and more immediate effect on ecological communities by quickly pushing organisms beyond their thermal optima (Stillman, 2019; Vasseur et al., 2014). Despite this, most experimental studies in freshwater ecology have so far applied static warming treatments in their design (Thompson et al., 2013; Woodward et al., 2016), generally set to predicted increase in annual mean temperatures by the end of the century (IPCC, 2023).

Changing temperature regimes can alter population dynamics and species interactions, leading to changes in community composition and ecosystem‐level processes (Ohlberger, 2013; Tylianakis et al., 2008). Aquatic species exhibit narrower thermal safety margins, that is, differences between organism's tolerance limits and the range of experienced temperatures in their habitats, compared to terrestrial taxa (Pinsky et al., 2019; Sunday et al., 2012). Consequently, they may be particularly vulnerable to sudden temperature changes, resulting in high community turnover (Comte & Olden, 2017). Effects of extreme temperatures may be especially strong in shallow lakes, where thermal refugia (e.g., hypolimnetic refuge) are often less prominent than in deep lakes (Martinsen et al., 2019).

The number of studies focusing on the effect of heatwaves on aquatic ecosystems is rising. Consequently, there is increasing evidence that heatwaves, often representing a short but intense disturbance, may limit the possibility for acclimation and adaptation and result in increased mortality and population declines (Till et al., 2019; Vad et al., 2023), shifts in species composition (Polazzo et al., 2022; Weisse et al., 2016), changes in species interactions (Li et al., 2017; Ross et al., 2022; Woodward et al., 2016), and may facilitate the growth of unwanted species or groups like cyanobacteria (Jöhnk et al., 2008; Wagner & Adrian, 2009). However, the majority of studies focusing on heatwaves applied single‐temperature treatments (e.g., Polazzo et al., 2022; Soulié et al., 2023; Vad et al., 2023), hindering our ability to forecast community dynamics under different climate change scenarios. The intensity and duration of heat disturbances can all determine the outcome of the effect of warming on aquatic communities (Seifert et al., 2015; Smale et al., 2015). Therefore, more studies utilizing comparative setups are needed to better understand how aquatic ecological communities respond to different warming scenarios, including the comparison of heatwaves and widely applied constant temperature setups.

Zooplankton play a key role in aquatic food webs by transferring energy from primary producers to higher trophic levels. Therefore, shifts in their dominant species or trait composition can severely alter energy flow in aquatic ecosystems (Hébert et al., 2016; Ye et al., 2013). By focusing on key traits, including morphological (body size, body mass, etc.), physiological (maximum growth rate, longevity, etc.), behavioral (feeding mode, escape responses, etc.), and life history traits (reproduction, clutch size, etc.) (Litchman et al., 2013), we can forecast functional community reorganization under warming scenarios and link these traits to changes in ecosystem functioning (Hébert et al., 2017; Litchman et al., 2013). A general morphological response to warming is a reduction in zooplankton body size (Brans et al., 2017; Moore & Folt, 1993), a trait that is linked to their grazing efficiency on primary producers (Gianuca et al., 2016; Schoenberg & Carlson, 1984). As body size is a master trait (Barton et al., 2013; Brown et al., 2004), if warming alters body size distribution (Evans et al., 2019; Moore & Folt, 1993), it can induce parallel changes in linked traits like body weight, as well (Sodré & Bozelli, 2019; Woodward et al., 2005). Physiological, behavioral, and life history traits can be similarly important when predicting the responses of zooplankton communities to environmental change. For example, physiological traits such as temperature optima are directly linked to species responses to warming and their population performance at different temperatures (Gao et al., 2015; Gerten & Adrian, 2002). Concerning life history traits, species with short generation time and fast population growth rate (e.g., parthenogenetically reproducing species) are expected to increase their densities or biomass more rapidly to increasing temperatures compared to, for example, sexually reproducing species with larval stages (Adrian et al., 2006; Allan, 1976; Johnsen et al., 2020). Larger omnivorous taxa, however, may increase predation pressure on small zooplankton and hence buffer heatwave effects (Zhang et al., 2018). In general, zooplankton, with a high diversity of traits and ecological strategies (Barnett et al., 2007) and their key position in aquatic food webs, provide an excellent model for understanding the mechanisms of community reorganization in response to different warming scenarios.

Trait‐based approaches are also powerful tools for addressing the mechanisms of ecosystem change beyond community shifts, including changes in food web interactions (Litchman et al., 2021; Litchman & Klausmeier, 2008). For example, decreasing body size of ectothermic organisms is a universal response to warming in freshwater systems at both population and community levels (Daufresne et al., 2009), which can weaken the strength of trophic cascades by reducing top‐down control (DeLong et al., 2015). On the other hand, a global assessment has found that warming can intensify trophic cascade effects on phytoplankton in food webs, suggesting that the role of trophic relationships across trophic levels will be particularly important under global environmental change (Su et al., 2021). Behavioral, morphological, physiological, and life history traits can all affect zooplankton feeding (Litchman et al., 2013). Community‐level shifts in key feeding‐related traits (such as motility, prey selection strategy, and grazing rate) can alter the magnitude and direction of energy flow (Litchman et al., 2021), eventually reflected in the provision of ecosystem services, for example, maintaining good water quality through top‐down control on algae (Declerck & de Senerpont Domis, 2022).

The aim of this study was to record and contrast the responses of zooplankton communities to warming treatments with constantly elevated mean temperatures versus heatwaves, and to link community‐level shifts in trait composition and functional groups to changes in the strength of top‐down control on phytoplankton. Warming treatment was designed with a constantly elevated average temperature compared to the ambient (control) treatment, whereas heatwave treatment was designed as recurring heat pulses applied over confined time periods over the experiment. Given the non‐linear relationship between a number of basic metabolic processes and temperature (Clarke, 2006), we expected that shorter, but more intense temperature disturbances (i.e., the heatwave treatment) would have a stronger and more immediate effect on community composition and ecosystem functioning. In line with this, we predicted that heatwaves would be followed by an increased dominance of fast‐growing parthenogenetic species with short generation times, which life history traits allow them to respond faster to sudden temperature changes. This may result in smaller community‐level body size, as populations of smaller‐sized species can generally achieve higher maximum growth rates (Nandini & Sarma, 2003). The dominance of smaller zooplankton species may imply weaker top‐down control on primary producers (Gianuca et al., 2016); hence, we expected increased phytoplankton biomass, especially in response to heatwaves.

## 2 MATERIALS AND METHODS

### 2.1 Experimental design and environmental parameters

We conducted a 1‐month long outdoor mesocosm experiment between June and July 2020 in the mesocosm system of Balaton Limnological Institute, Centre for Ecological Research, Hungary (N 46°54′48.5″, E 17°53′33.9″). The twelve mesocosms were filled directly with water from Lake Balaton 2 days before the start of the experiment. We filled them from the shallow, littoral zone through a hose, where the effect of water stratification can be considered negligible. The water level was set to 1.20 m, resulting in an experimental volume of 3000 L (inner diameter of mesocosms: 2 m; maximum depth: 1.5 m). The water column of each mesocosm was constantly mixed with an airlift system (with 0.6 m^3^ h^−1^ carrying capacity) to prevent vertical stratification (Striebel et al., 2013), hence representing shallow lake ecosystems that are regularly mixed by the wind. Besides, it also ensured that dissolved oxygen levels were saturated in all mesocosms during the experiment, which was followed with daily DO measurements with a sensor (PONSEL manufacturer). The airlift consisted of a PVC pipe hanging in the center of each mesocosm, in which compressed air released from a tube produced a gentle upward current. As the primary focus of the experiment was to study the responses of pelagic plankton communities, we did not include fish, macrophytes, or sediment in the mesocosms. Mesocosms were covered with a mosquito mesh to prevent larger debris from falling into the tanks and the colonization of macroinvertebrates that could influence the nutrient level and species interactions in the water.

**FIGURE 1:**
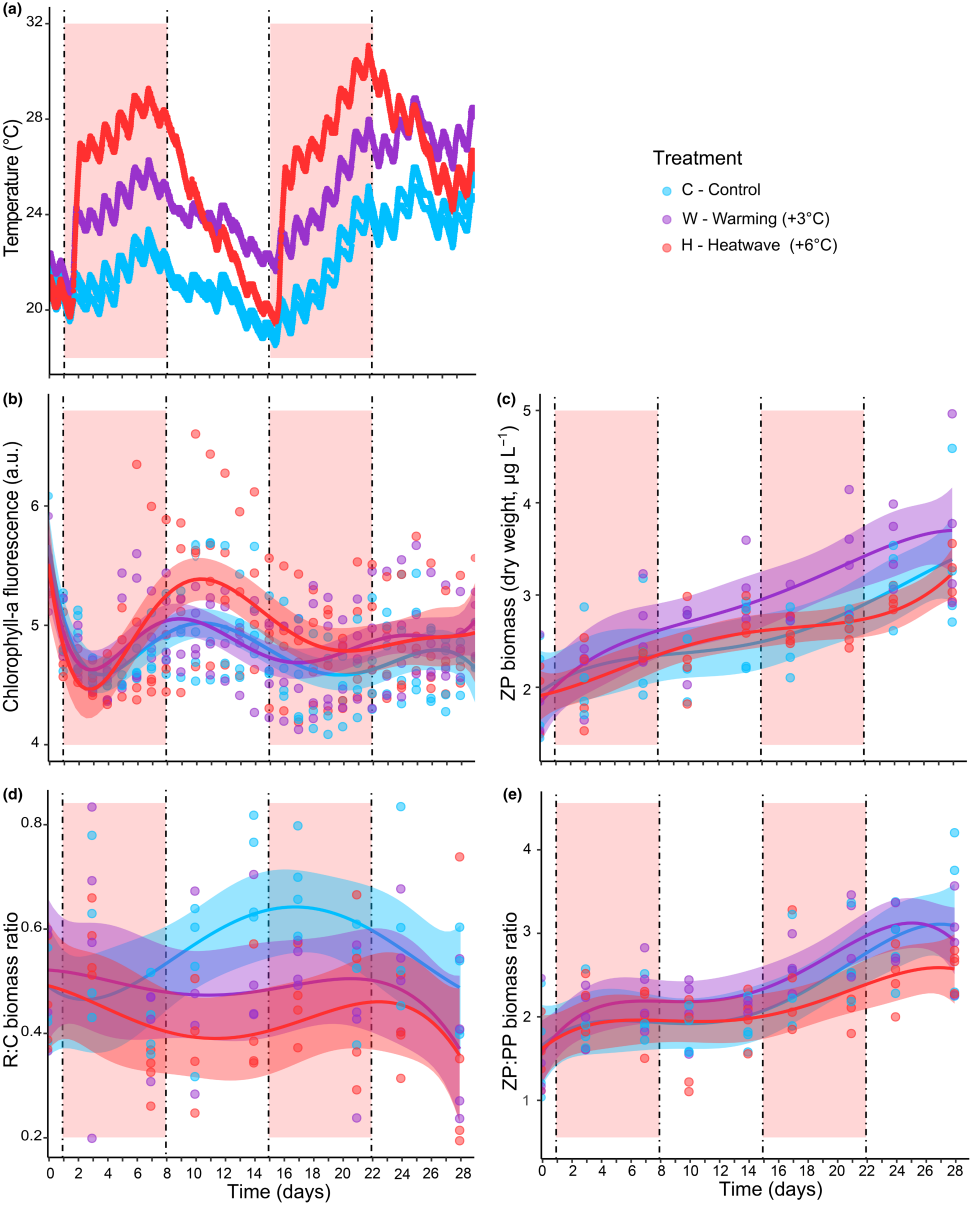
Temporal patterns in (a) observed water temperature (°C), (b) log‐transformed chlorophyll *a* (Chl*a*) fluorescence (proxy of phytoplankton biomass) (a.u.), (c) zooplankton (ZP) biomass (dry weight, μg L^−1^), (d) biomass ratio of Rotifera (R) to Copepoda (C), and (e) biomass ratio of total zooplankton (ZP) to phytoplankton (PP) in the three (color‐coded) treatments. Biomass data were double‐square‐root transformed for the analyses. Solid trend lines and error bands represent fitted GAMs ± SE. Red backgrounds indicate the lengths of the two experimental heatwaves (H).

Three treatments, each replicated four times, were randomly assigned to the 12 mesocosms: (1) ambient environmental conditions (C—control); (2) warming set to a constant elevation of 3°C above the control conditions (W—warming); and (3) recurring 1‐week heatwaves (H—heatwave) with temperature increased by 6°C above the control conditions in the first (between day 1 and day 8) and the third weeks (between day 15 and day 22) (Figure 1a). Both warming treatments (W and H) received an identical total energy input but in different pulses, and both started on the first day of the experiment. The rate of temperature increases in the W treatment aimed to simulate a moderate rise (+3°C) in water temperature by the end of the century (IPCC, 2023), while H was designed with the same energy input but two more intense pulses. A programmable logic controller (PLC) system recorded water temperature automatically in 10‐min intervals and at the same time‐controlled water temperature in the heated tanks. The average temperature values of the four control mesocosms were used as a baseline to set temperatures in the warming treatments. Regular nutrient measurements were carried out to monitor and, if necessary, ensure relatively constant levels of basal resources by nutrient addition, which was eventually not necessary as they were comparable across treatments (Figure S1).

### 2.2 Nutrient measurements

To obtain data on nutrient concentrations over the experimental duration, we took samples twice a week on the same dates when collecting the zooplankton samples (days 0, 3, 7, 10, 14, 17, 21, 24, and 28). A vertically integrated water sample was collected with a PVC tube sampler and pooled from three random locations in each mesocosm. Total nitrogen (TN) concentration was determined with a Multi N/C 3100 TC‐TN analyzer. Concentrations of inorganic nitrogen compounds, including nitrate (NO_3_^−^) and ammonium (NH_4_^+^), were measured according to standard spectrophotometric methods (Eaton et al., 2005). For the determination of soluble reactive phosphorus (SRP), water samples were first filtered on a regenerated cellulose membrane filter (Whatman RC 55, 0.45 μm pore size), then concentrations were quantified according to standard spectrophotometric methods (Eaton et al., 2005). Total phosphorus (TP) concentrations of unfiltered water samples were measured with the same method described for SRP but following persulfate acidic digestion.

### 2.3 Chlorophyll *a*

The same water sample (vertically integrated, pooled from three locations) used for nutrient measurements was also used to determine chlorophyll *a* (Chl*a*) concentrations. We first filtered the water sample through a plankton net (100‐μm mesh size to obtain water free of large zooplankton. Fresh samples were regularly checked for overly large filaments and colonies, but none of these were abundant in mesocosms. To measure Chl*a* concentration, a proxy for phytoplankton biomass, 500–850 mL of water (depending on algal densities) was filtered through glass microfiber filters (Whatman GF/F) twice a week. Subsequently, the spectrophotometric determination was carried out after hot methanol extraction, using the absorption coefficients determined by Iwamura et al. (1970) and the original water volume filtered. On average, 1% of dry weight can be accounted for by chlorophyll *a* (Reynolds, 2006). Therefore, data was converted to phytoplankton dry weight (dry weight, μg L^−1^) by a multiplication factor of 100 for subsequent analyses (Reynolds, 2006).

Additionally, to obtain higher‐frequency data on Chl*a*, and hence phytoplankton dynamics, we measured Chl*a* fluorescence daily (maximum fluorescence yield; Figure 1b) with a handheld fluorometer (AquaPen AP 110‐C, Photon System Instruments). We took samples from each mesocosm every morning during the experiment from the central surface water of the mesocosms (next to the airlift, where water was well‐mixed). Measurements were carried out after a 30‐min dark adaptation period to avoid potential bias resulting from short‐term physiological changes (Perri et al., 2021). Chl*a* fluorescence was measured using the OJIP protocol (Stirbet & Govindjee, 2011). We assessed the overall precision of Chl*a* fluorescence measurements by testing the relationship between Chl*a* concentration and fluorescence via linear regression (Figures S2 and S3).

### 2.4 Zooplankton

To determine initial densities and community composition, zooplankton were sampled at the start of the experiment (day 0, before the start of the warming treatments). Subsequently, samples were collected twice a week with a Schindler‐Patalas plankton trap (volume: 10 L, with a net mesh size: 45 μm) after thorough mixing of the water column with a stick. We collected zooplankton samples from three randomly chosen locations of the mesocosms, which represented different vertical layers, hence resulting in a depth‐integrated sample. We preserved zooplankton samples in 70% ethanol. We counted and identified zooplankton with an inverted microscope at 20× magnification. For microcrustaceans, we used the average body lengths obtained from the regular monitoring data of Lake Balaton (provided in Table S1), and then we converted body length to biomass (dry weight), following published length–weight relationships (McCauley, 1984). As similar reference data were not available for Rotifera, we calculated the average body length of each species based on literature data (Cieplinski et al., 2017; Gosse, 1851; Roche, 1993; Skorikov, 1986), and biomass (dry weight) was calculated based on length‐weight relationships (Finlay & Uhlig, 1981).

Traits were assigned to each species: body length, body mass, reproduction mode, feeding mode, generation time, and clutch size, according to Table S1. These represent an exhaustive list of the most common morphological and behavioral traits (except for escape response traits, as the mesocosms did not contain any potential predators of zooplankton), as well as life history traits found and used in the literature. We did not use physiological traits as they were not available for all the species. We measured the body length of adult male and female individuals of the two copepod species, *Mesocyclops leuckarti* and *Eudiaptomus gracilis* after the second heatwave (day 24) in all three treatments (min. 10% of all individuals per sample), in order to screen for potential intraspecific responses to the treatments in terms of body size (Figure S4).

### 2.5 Biomass ratios as indices for changes in top‐down control

The biomass ratio of organisms at higher and lower trophic positions can be used as a measure of the strength of top‐down control (Shurin et al., 2012). We, therefore, calculated the ratio between zooplankton (ZP, dry weight, μg L^−1^) and phytoplankton (PP, dry weight, μg L^−1^) biomass, referred to as ZP:PP, and tested its potential response to the different warming treatments. As a further measure of changes in the trophic structure and functioning, we also analyzed the temporal trend of the biomass ratio of the two dominant functional groups, the small‐bodied grazer Rotifera and the larger omnivorous Copepoda across treatments (R:C).

### 2.6 Data analysis

To visualize the temporal dynamics of Chl*a*, zooplankton biomass (ZP), and ratios of zooplankton: phytoplankton (ZP:PP) and Rotifera:Copepoda (R:C) in the different treatments, we fitted generalized additive models (GAMs) on the respective data for each treatment over the experimental duration. For this, we used the “geom_smooth” function of the package “ggplot2” (Wickham, 2016), with the number of knots (*k*) *k* = 6 for Chl*a* and *k* = 4 for the other datasets based on the “gam. check” function in the package “mgcv” (Wood, 2017). To test for treatment‐specific differences for ZP, ZP:PP, and R:C, we performed non‐parametric Kruskal–Wallis (KW) tests for each sampling date. Subsequently, we applied Dunn's post hoc test to reveal pairwise differences (*p*‐values were adjusted with the Holm method) using the package “FSA” (Ogle et al., 2023). The same tests were carried out for the concentration of nutrients. The results of these analyses are presented in Table S2.

To test the effect of treatments on daily Chl*a* fluorescence, we used a generalized additive mixed model (GAMM) with treatment as the main linear predictor, adding time with varying shapes of smooth according to individual mesocosms and a temporal autocorrelation within individual mesocosms to account for random effects. As Chl*a* still showed a decreasing trend during the first 4 days (days 0–3) in all treatments, likely linked to filling up the mesocosms (e.g., ongoing sedimentation), we excluded this period from the analysis. Additionally, we ran a Kruskal–Wallis test to assess whether there was a significant difference between the treatments in this 4‐day initial period (Figure S5), where we did not find a significant treatment effect. As we did not find a significant difference based on the GAMM when analyzing the dataset encompassing days 4–28 (Table S3), we split the dataset into two periods: days 4–14 including the first heatwave (until day 7) and its direct aftermath (days 8–14), and days 15–28 including the second heatwave (days 15–21) and its direct aftermath (days 22–28). In case of a significant treatment effect based on the GAMM, we performed a pairwise test for multiple comparisons with single‐step *p*‐value adjustment using the package “multcomp” (Bretz et al., 2010).

To test how environmental predictors, or in our case, treatments, can filter species traits based on species abundances, we performed RLQ analyses (Dolédec et al., 1996) with the package “ade4” (Dray & Dufour, 2007) separately for each date. The RLQ method performs a double inertia analysis including three data matrices (environmental variables by samples—R; species by samples—L table; and traits by species—Q table) and produces their simultaneous ordination. Fourth‐corner statistics were computed based on 9999 permutations by permuting both sites and species (with “modeltype = 6”), to avoid Type I error (Dray et al., 2014). We used the log(*x* + 1) transformed biomass of zooplankton species and the traits presented in Table S1.

To quantify the direct and indirect trophic relationships between the main organism groups during the first experimental period, when there was a significant effect of H on Chl*a* fluorescence, we applied structural equation models (SEM) on the copepod and rotifer biomasses and Chl*a* fluorescence. We excluded Cladocera as their abundances and biomasses were very low compared to the other zooplankton groups, being absent in many samples (Figures S6–S9). We started with an initial SEM that included all the plausible pathways between plankton groups and the treatments using the R package “piecewiseSEM” (Lefcheck et al., 2015). Each relationship within the SEM was estimated with linear mixed‐effects models with a temporal autocorrelation structure of order 1 (AR‐1) and mesocosm ID as a random factor. To meet the model assumptions (normality of residuals, heterogeneity of variances), double square‐root transformation was applied for the copepod, and cubic‐root transformation was applied for the rotifer data. We expected that any top‐down effects on phytoplankton would manifest with a time lag. Therefore, separate SEMs were fitted using the Chl*a* values on the zooplankton sampling days and the values 0–6 days after the samplings. The SEM yielding the highest explanatory power for Chl*a* (based on *R* ^2^) was selected to describe the causal network between the three organism groups and the treatments. Similar SEMs were run for the same experimental period for W, and both for treatments H and W in the second experimental period (i.e., the second 2 weeks of the experiment; Table S4). Additionally, we compared the magnitude of the direct and indirect effects of the first experimental heatwave on phytoplankton biomass based on different time lags. Indirect effects were calculated by multiplying the path coefficient for the effect of the H treatment on Rotifera biomass with the path coefficient for the effect of Rotifera biomass on phytoplankton biomass. All statistical analyses were performed using R version 4.0.2 (R Core Team, 2020).

## 3 RESULTS

Temperature varied between 18.5–26.0°C in C, 18.9–28.9°C in W, and 19.4–31.1°C in H during the 4 weeks of the experiment (Figure 1a, Figure S10). We found significantly higher Chl*a* fluorescence in treatment H than in W and C in the first period of the experiment, including the first heatwave (days 4–14; GAMM with pairwise tests, H‐C estimate: 0.27, *p* = .001; H‐W estimate: 0.24, *p* = .005; Table S3). Differences were not significant in the second experimental period (days 15–28; GAMM with pairwise tests, H‐W estimate; 0.04, *p* > .05; H‐C estimate: 0.19, *p* > .05, W‐C estimate: 0.15, *p* > .05; Table S3). A peak in Chl*a* fluorescence was observed in treatment H after the first heatwave (Figure 1b). Nutrients showed no significant response to the treatments in the first 2 weeks, and even in the second 2 weeks, we only detected a single occasion (day 17) when SRP was higher in the W than in the C treatment (Table S2).

In our experiment, the copepods *Mesocyclops leuckarti* and *Eudiaptomus gracilis* and the rotifer *Polyathra remata* dominated zooplankton by accounting for 19.0 ± 24.6, 13 ± 9.8, and 4 ± 6.6% (mean ± SD, calculated from all data available for the whole experiment) of the total biomass over the experiment (Figures S11 and S12). We found an overall increase in zooplankton biomass over time with similar trends across treatments (Figure 1c). Especially towards the end of the experiment, biomass in the W treatment showed a trend of becoming higher than in the other two treatments, but a significant difference was only found between W and H treatments (KW test: chi^2^ = 7.65, *df* = 2, *p* = .02, W‐H: *p* < .01) at the end of the second heatwave (day 21, Table S2). Overall, the R:C biomass ratio decreased significantly as a response to H compared to the C treatment. The decrease of R:C biomass ratio became evident after the first heatwave (day 14; KW test: chi^2^ = 7.04, *df* = 2, *p* = .03, H‐C: *p* < .01) and lasted until the first part of the second heatwave (day 17; KW test: chi^2^ = 8.35, *df* = 2, *p* = .02, H‐C: *p* < .01; Figure 1d, Table S2). We found an increasing trend in the ZP:PP biomass ratio with significant differences between W and H at the end of the second heatwave (day 21; KW test: chi^2^ = 4.77, *df* = 2, *p* = .09, H‐W: *p* = .03, Figure 1e, Table S2). We did not find any population‐level body size responses to the treatments in the dominant copepods (Dunn's post hoc tests: *p* > .05; Figure S4).

**FIGURE 2:**
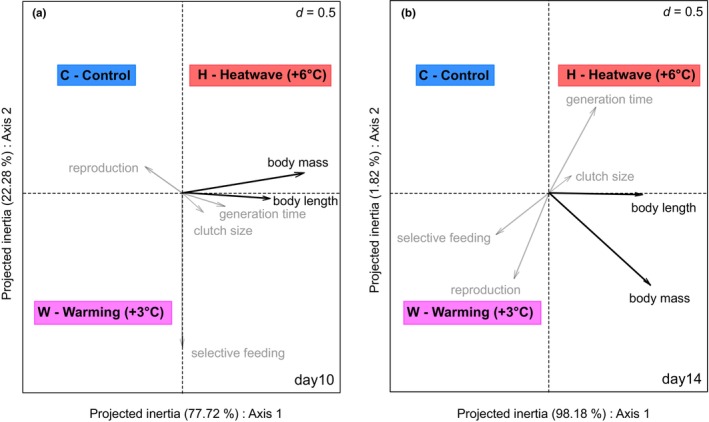
RLQ plots for the relationships between zooplankton traits and experimental treatments after the first heatwave on days 10 (a) and 14 (b). Traits having at least one significant (*p* < .05) relationship with at least one treatment based on fourth‐corner analyses are shown with black arrows, while others are in gray. (Grid size: D = 0.5).

We found significant relationships between traits and treatments revealed by fourth‐corner analysis (related RLQ plots presented in Figure 2), with body‐size related traits (body mass, body length) responding positively to H. Specifically, body mass (day 10, *p* = .048) and body length (day 10, *p* = .046) were positively associated with H in the period after the first heatwave (Figure 2). At the same time, both traits were negatively associated with the control treatment on day 14 (body mass: *p* = .046; body length: *p* = .031). During and after the second heatwave, we did not find any significant treatment effects (Figure S13).

**FIGURE 3:**
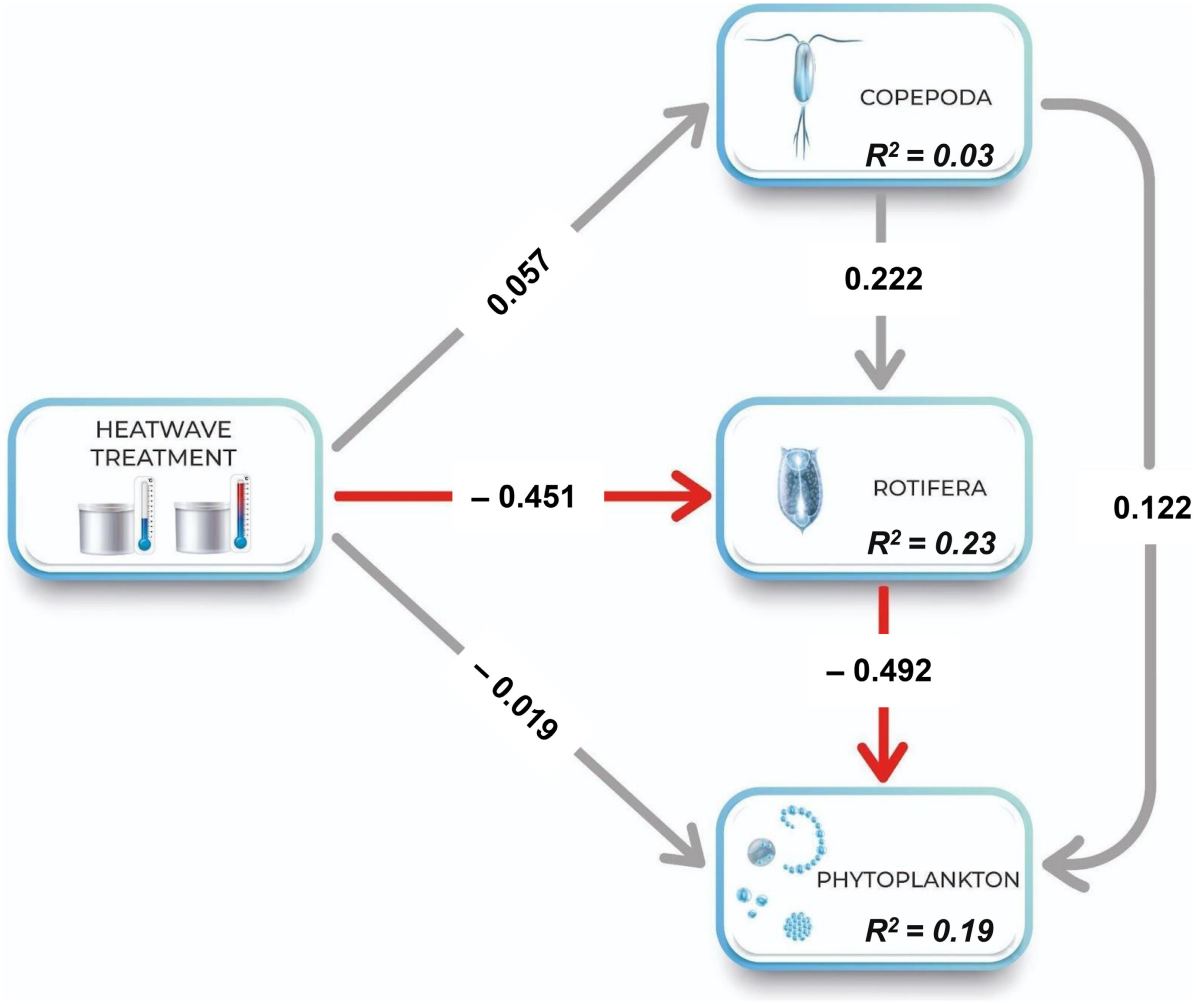
A structural equation model (SEM) of the linkages between the dominant zooplankton groups, Rotifera and Copepoda (biomass), and phytoplankton (Chl*a* fluorescence) in the heatwave treatment during the first 2 weeks of the experiment. The model presented here is based on a time lag of 4 days between Chl*a* and zooplankton when the marginal R^2^ shows the highest value among the time lags. Solid red arrows represent direct, significantly negative pathways (*p* < .05), while gray arrows stand for non‐significant direct pathways. Numbers represent standardized parameter estimates. Marginal R^2^s for component models are given in the boxes of endogenous variables. The amount of variation explained by the model (along with others with different time lags) is presented in Table S4 and Figure S14.

Treatment H exerted a direct and significant negative effect on Rotifera biomass based on the SEM (standardized beta estimate: −0.451, *p* < .05, Figure 3, Table S4). Besides, the SEM revealed a direct negative effect of Rotifera on Chl*a* fluorescence, which appeared with a time lag of 2–5 days (Figure 3, Table S4). The relative strength of the direct to indirect effects of the treatment on Chl*a* depended on the actual day used as the time lag (Figure S14), with more comparable magnitudes with a time lag of, for example, 2 or 3 days (at the same time, these days were not the ones with the highest explanatory power for Chl*a*). We did not find any significant effects of W treatment on the biomass of Copepoda, Rotifera, or phytoplankton (Table S4).

## 4 DISCUSSION

The results of this study supported our expectation that heatwaves with shorter but more intense temperature increases exert stronger and more immediate effects on plankton community composition compared to constantly elevated temperatures with equivalent energy input. However, contrary to our expectation, heatwaves had a negative impact on the biomass of small‐bodied zooplankton (Rotifera) in our study. This result has important implications for ecosystem functioning, as the decline in small‐bodied grazers, in turn, resulted in reduced top‐down control of phytoplankton and contributed to elevated algal biomass during the first heatwave.

The positive association between body size and heatwaves, revealed by the RLQ analysis, was counterintuitive as most existing studies reported zooplankton body size to decrease with warming, spanning from population to community levels (Brans et al., 2017; Brucet et al., 2010). However, these studies largely derived their conclusions from natural temperature gradients (where species can adapt over time) or experiments with constant warming setups. Heatwave effects, in contrast, may be less predictable due to stronger pressure on individual physiology, potentially compromising physiological or genetic adaptations (Dam & Baumann, 2017), resulting in increased mortality rates (Stillman, 2019), or sudden changes in species interactions and phenology (Zhang et al., 2018). In our study, the positive association between heatwaves and larger body size resulted from the decrease of microzooplankton (Rotifera) biomass, which overall shifted the communities to larger body size (resulting from Copepoda dominance) in the H treatment.

The decline in Rotifera biomass may be a direct effect of the heatwave causing thermal stress, which can lead to decreased survival and reproduction (Paraskevopoulou et al., 2020). At the same time, the most dominant Rotifera was *Polyarthra remata* (Figure S12), a eurythermic summer species (Bērzinš & Pejler, 1989). This indicates the potential relevance of indirect effects, that is, mediated by food web interactions, such as increased predation by copepods. Many species of freshwater cyclopoid and calanoid copepods, including the dominant species of our study, *Eudiaptomus gracili*s, and *Mesocyclops leuckarti* (Figure S11), are known to be predators of rotifers (Brandl, 2005; Hansen & Santer, 1995; Šorf & Brandl, 2012; Williamson, 1983), and often feed preferentially on rotifers over phytoplankton (Hansen & Santer, 1995; Kunzmann et al., 2019; Williamson & Butler, 1986). We did not observe a negative correlation between the biomass of Copepoda and Rotifera. It still may be possible that the increased metabolic demand of Copepoda during the heatwaves resulted in higher predation rates on Rotifera, eventually leading to their biomass decline. This does not necessarily result in any changes in the biomass of ectothermic consumers (in our case, Copepoda), as more energy is required to maintain similar biomass levels at higher temperature levels, which can be covered by increased food uptake (Brown et al., 2004; Zhang et al., 2020). The fact that the relationship between Copepoda and Rotifera biomass changes from positive to no significant relationship in the H treatment without any considerable change in Copepoda biomass further supports that the loss of Rotifera biomass was not directly driven by increased Copepoda biomass (Figure S15).

Increased algal biomass as a response to warming and heatwaves is a commonly observed phenomenon, which may result from direct and indirect (i.e., food web) effects (Ger et al., 2016; Viitasalo & Bonsdorff, 2022). A moderate increase in temperature increases both the phytoplankton growth rate and the metabolic demands of consumers, leading to higher feeding rates and, consequently, higher consumer‐to‐producer biomass (Kratina et al., 2012; O'Connor et al., 2009). Heatwaves, at the same time, may lead to the opposite pattern by reducing the strength of top‐down control and consequently increasing the biomass of phytoplankton through a negative impact on their key zooplankton grazers, such as *Daphnia* (Vad et al., 2023). In our experiment, zooplankton was dominated by Copepoda and Rotifera, while large‐bodied Cladocera, such as *Daphnia*, were rare. This has important implications for food web dynamics in our experimental system given the known predator–prey interactions between Copepoda and Rotifera (Williamson, 1983; Zhang et al., 2018). The food preference of planktonic predators, such as copepods, can strongly affect herbivore composition and abundance, which can thus indirectly shift size distribution within the phytoplankton (Ehrlich & Gaedke, 2020). In contrast to cladocerans, copepods exert a stronger top‐down control on microzooplankton and (Adrian & Schneider‐Olt, 1999; Williamson, 1987) the larger fraction of phytoplankton (Sommer et al., 2001; Sommer & Sommer, 2006). Therefore, small‐sized phytoplankton can benefit from reducing grazing pressure (Sommer & Sommer, 2006), which was most likely an important mechanism in our experiment as well, where the algal biomass peaks of the H treatment were dominated by small‐celled *Chlorella* (mean size: 4.5 μm; mean relative biomass: 65.0%) and *Monoraphidium* species (mean size: 6.5 μm; mean relative biomass: 22.8%) (K. Pálffy, pers. obs.). Since elevated phytoplankton biomass appeared without external nutrient input (TP concentration was similar among treatments), it likely resulted mostly from indirect effects through zooplankton (see also Figure S14).

An intriguing observation of our study is that the heatwave effect on phytoplankton biomass occurred only after the first heatwave. This effect diminished later on, even though the second heatwave peaked at higher temperatures. It is unlikely that the lack of phytoplankton peaks in the second half of the experiment was related to grazing by Rotifera, as they did not recover in the heatwave treatment (Figure S9). A possible explanation is that copepods, with steadily increasing biomass over time (Figure S7), performed a diet shift to the more abundant food sources, that is, phytoplankton over Rotifera (Kiørboe et al., 1996). Omnivorous copepods can become more herbivorous with increasing temperature (Boersma et al., 2016), representing another possible explanation for the absence of higher phytoplankton biomass during the second heatwave.

Mesocosm experiments are useful tools to identify community‐ and ecosystem‐level shifts to climate change by representing a compromise between experimental control and realism (Fordham, 2015; Stewart et al., 2013). In our study, the lack of top‐down control due to the absence of higher trophic levels (e.g., fish, macroinvertebrates) likely explains the temporal increase in zooplankton biomass in all treatments. However, by this reduction of trophic complexity, planktonic community shifts and food web interactions could be revealed without confounding factors. Though the 1‐month duration of our study limits long‐term forecasts, it delivers relevant information on the short‐term effects of intermittent heatwaves on community dynamics. The heatwave treatment, which represented more intense heat disturbances for shorter periods, had a stronger effect on communities than the constant warming treatment with less warming intensity. This result has important implications for freshwater communities as more intense and longer heatwaves are predicted in the future (IPCC, 2023; Woolway et al., 2021). It is also important to mention that the lack of strong effects in the constant warming treatment may also be related to the relatively short experimental duration. It represented a less intense, but permanent disturbance, which may only have an effect on longer time scales, in line with the concept of press disturbance (Bender et al., 1984; Glasby & Underwood, 1996).

Here, we showed that heatwaves could exert a stronger short‐term pressure on planktonic communities than a long‐term continuous warming treatment. Most importantly, we showed how heatwave‐driven planktonic interactions indirectly lead to increased algal biomass. At the same time, the lack of lasting effects at this temporal scale implies that communities in shallow lakes may be, to some extent, resilient to such short‐term heat perturbations. As natural systems are facing heatwaves of increasing magnitude and duration, stronger effects on communities and ecosystems are expected in the future. Longer‐term studies are required to be able to create more accurate predictive models and thereby improve our forecasting ability, while experimental studies should incorporate temperature fluctuations besides focusing on the predicted increase in mean temperature.

## AUTHOR CONTRIBUTIONS

**Thu‐Hương Huỳnh:** Data curation (lead); formal analysis (lead); investigation (supporting); methodology (lead); writing – original draft (lead); writing – review and editing (lead). **Zsófia Horváth:** Conceptualization (equal); data curation (lead); formal analysis (lead); funding acquisition (supporting); methodology (lead); supervision (equal); writing – original draft (lead); writing – review and editing (lead). **Károly Pálffy:** Formal analysis (lead); writing – review and editing (supporting). **Vivien Kardos:** Investigation (equal); supervision (supporting); writing – review and editing (supporting). **Beáta Szabó:** Data curation (supporting); formal analysis (supporting); investigation (supporting); visualization (supporting); writing – review and editing (supporting). **Péter Dobosy:** Investigation (supporting); methodology (lead); writing – review and editing (supporting). **Csaba F. Vad:** Conceptualization (equal); data curation (equal); funding acquisition (equal); investigation (lead); methodology (lead); supervision (lead); writing – original draft (lead); writing – review and editing (lead).

## CONFLICT OF INTEREST STATEMENT

The authors declare no conflicts of interest.

## Supporting information


Appendix S1

Appendix S2

## Data Availability

Data will be made available in the Dryad data repository.
