# Supplementary material for: Heatwave‐induced functional shifts in zooplankton communities result in weaker top‐down control on phytoplankton
Source: Ecol Evol. 2024 Aug 6;14(8):e70096. doi: 10.1002/ece3.70096 (PMC11300956; doi:10.1002/ece3.70096)
Supplement: Supplementary file 1 — Appendix S1 [file ECE3-14-e70096-s002.docx]

**Supplemental Information**

**Title: Heatwave-induced functional shifts in zooplankton communities result in weaker top-down control on phytoplankton**

**Running head: Climate change effect on plankton communities**

Thu-Hương Huỳnh^1,2,3^, Zsófia Horváth^1,3^, Károly Pálffy^1,3^, Vivien Kardos^1^, Beáta Szabó^1.3^, Péter Dobosy^1^ & Csaba F. Vad^1,3^

^1^Institute of Aquatic Ecology, HUN-REN Centre for Ecological Research, Budapest, Hungary

^2^Doctoral School of Biology, Institute of Biology, Eötvös Loránd University, Budapest, Hungary

^3^National Multidisciplinary Laboratory for Climate Change, HUN-REN Centre for Ecological Research, Budapest, Hungary

**Corresponding author**

Thu-Hương Huỳnh, Institute of Aquatic Ecology, Centre for Ecological Research, Budapest, Karolina út 29, 1113 Budapest, Hungary.

Email: [huynh.huong@ecolres.hu](mailto:huynh.huong@ecolres.hu)

**
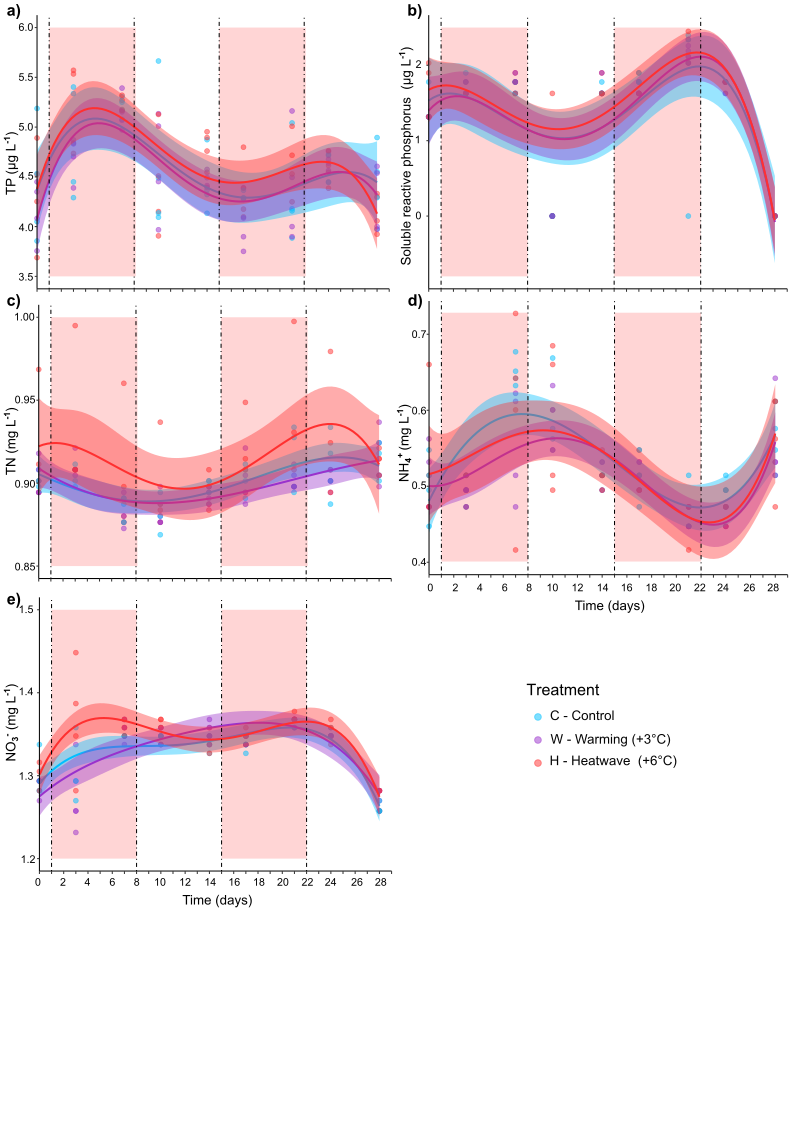
**

**Figure S1.** Temporal patterns of nutrient concentrations during the experiment. All concentrations were double-square-root transformed before analysis. Solid trend lines and error bands represent fitted GAMs ± SE. Red backgrounds indicate the periods of the two experimental heatwaves.


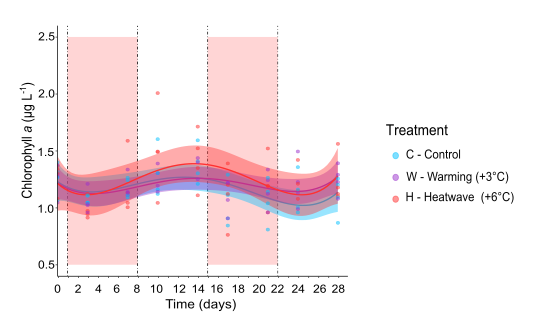


**Figure S2.** Dynamics of chlorophyll *a* (µg L^-1^, based on pigment extraction) over the experiment. Solid trend lines and error bands represent fitted GAMs ± SE. Red backgrounds indicate the two experimental heatwave periods.

**
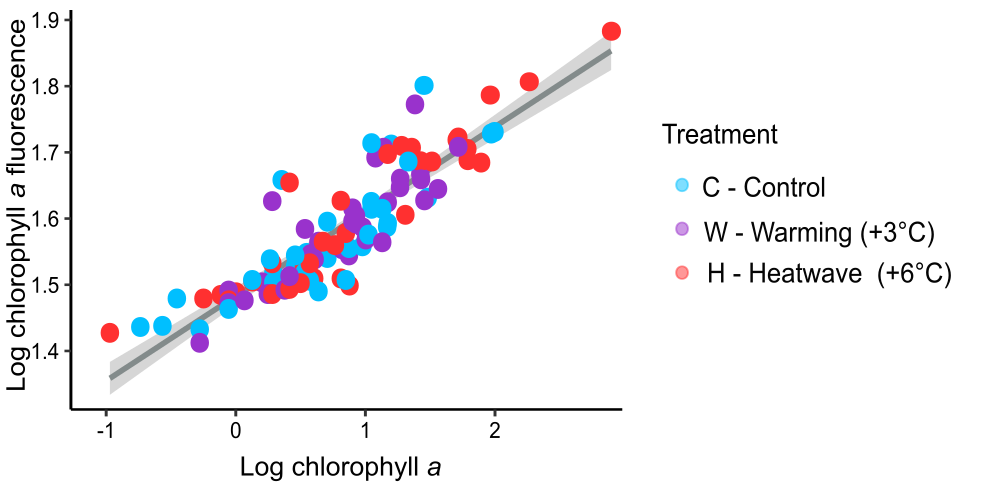
**

**Figure S3.** Linear regression between spectrophotometrically determined chlorophyll *a* (Chl*a*) concentration and fluorescence measured with a hand-held fluorometer (R^2^=0.8). Chl*a* concentration data were measured on 9 days during the experiment (see **Figure S2**), and these data were plotted against the respective Chl*a* fluorescence data (N=108).


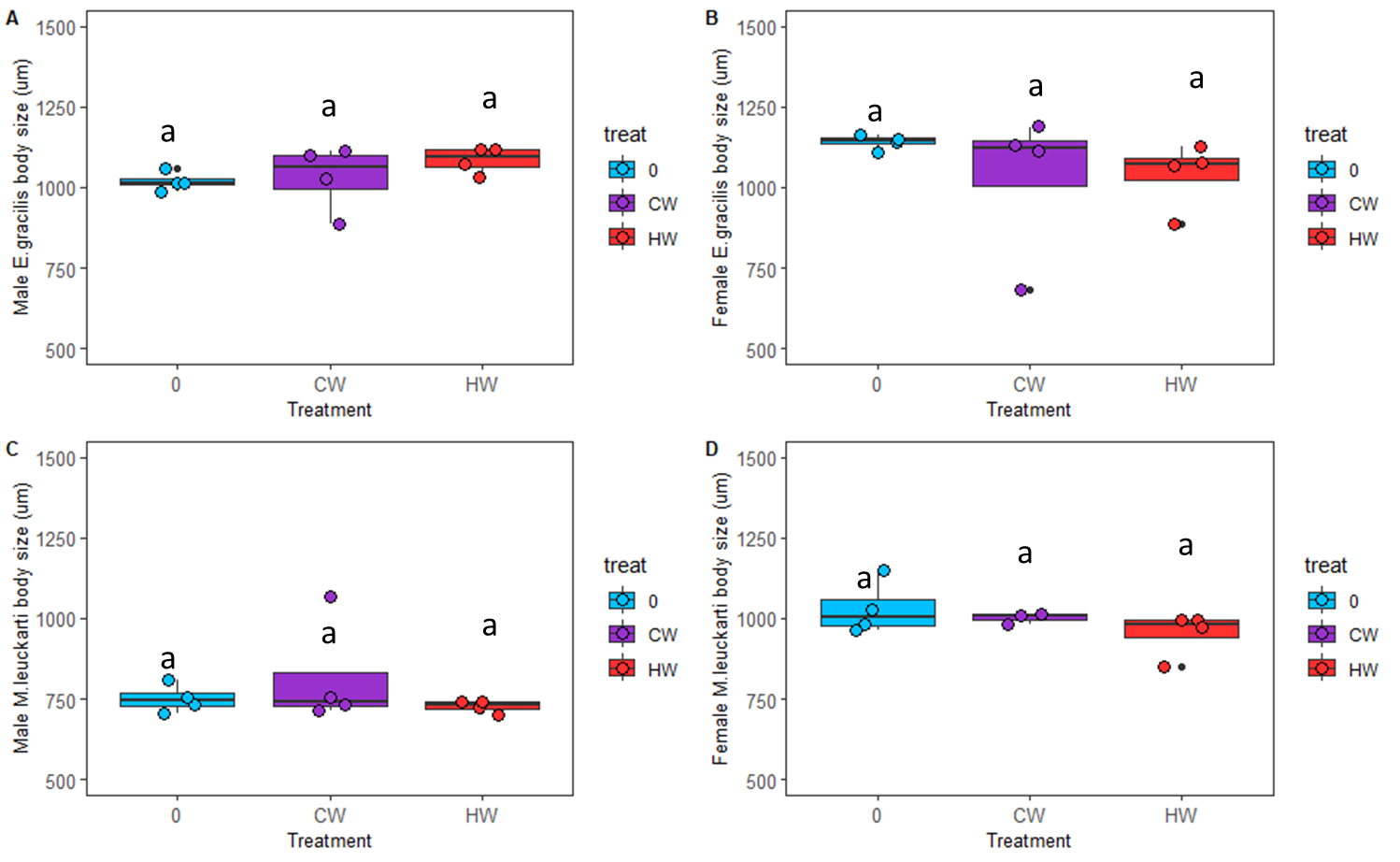


**Figure S4.** Boxplots illustrating the zooplankton body size (µm) (of **(a)** Male *Eudiaptomus gracilis*, **(b)** Female *Eudiaptomus gracilis*, **(c)** Male *Mesocyclops leuckarti*, and (d). Female *Mesocyclops leuckarti* right after the second heatwave (day 24). N = 4 for each treatment. No significant differences (indicated by letters) were found among treatments based on Kruskal–Wallis (KW) tests and Dunn's *post hoc* test applied to reveal pairwise differences.


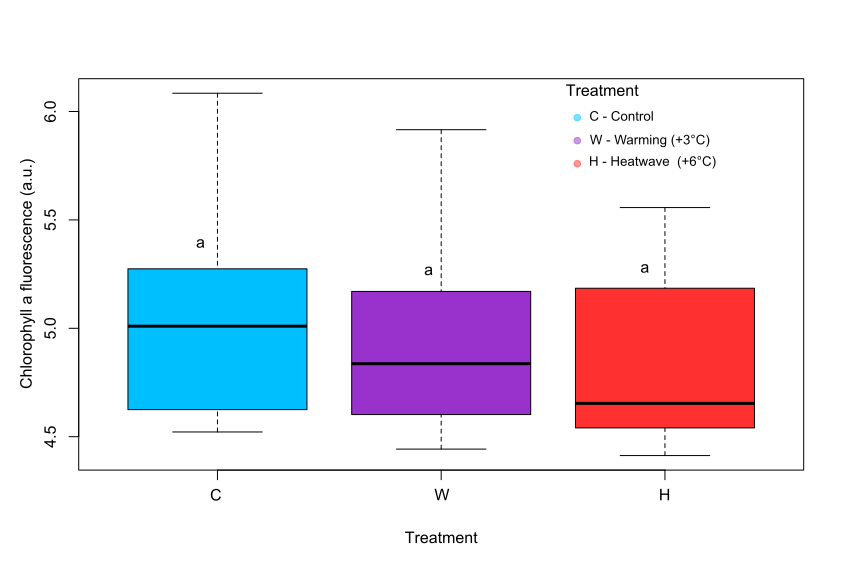


**Figure S5.** Boxplots illustrating chlorophyll *a* fluorescence (a.u.) in the different treatments from day 0 to 3 (N=16 for each treatment). No significant differences (indicated by letters) were found among treatments based on Kruskal–Wallis (KW) tests and Dunn's *post hoc* test applied to reveal pairwise differences.


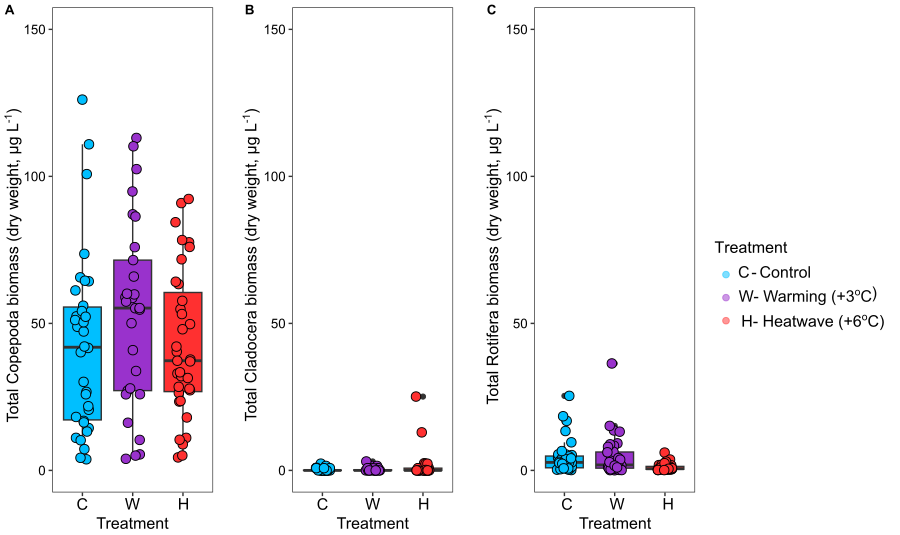


**Figure S6.** Boxplots illustrating the biomass (dry weight, µg L^-1^) of **(a)** Copepoda, **(b)** Cladocera, and **(c)** Rotifera based on data collected throughout the experiment (N=36 for each treatment).

**
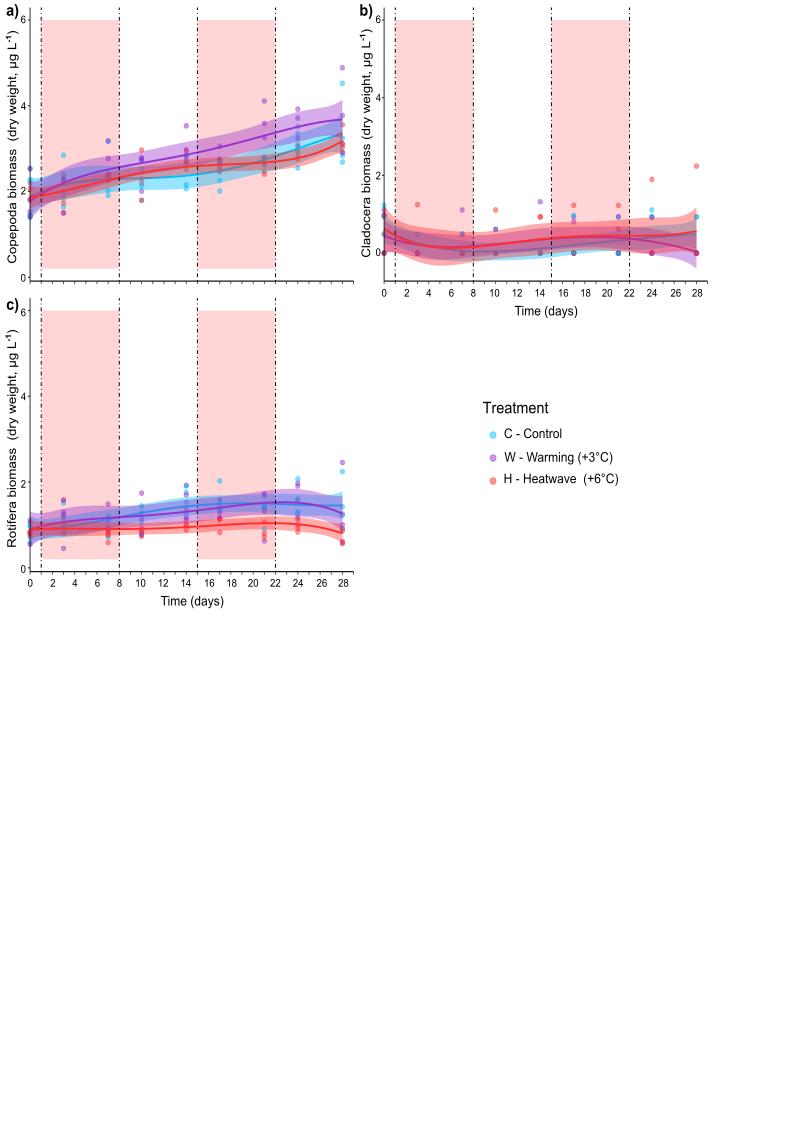
**

**Figure S7.** The temporal dynamics in biomass (dry weight, µg L^-1^) of **(a)** Copepoda (data are double-square-root transformed), **(b)** Cladocera, and **(c)** Rotifera during the experiment. Solid trend lines and error bands represent fitted GAMs ± SE. Red backgrounds indicate the lengths of the two experimental heatwaves.


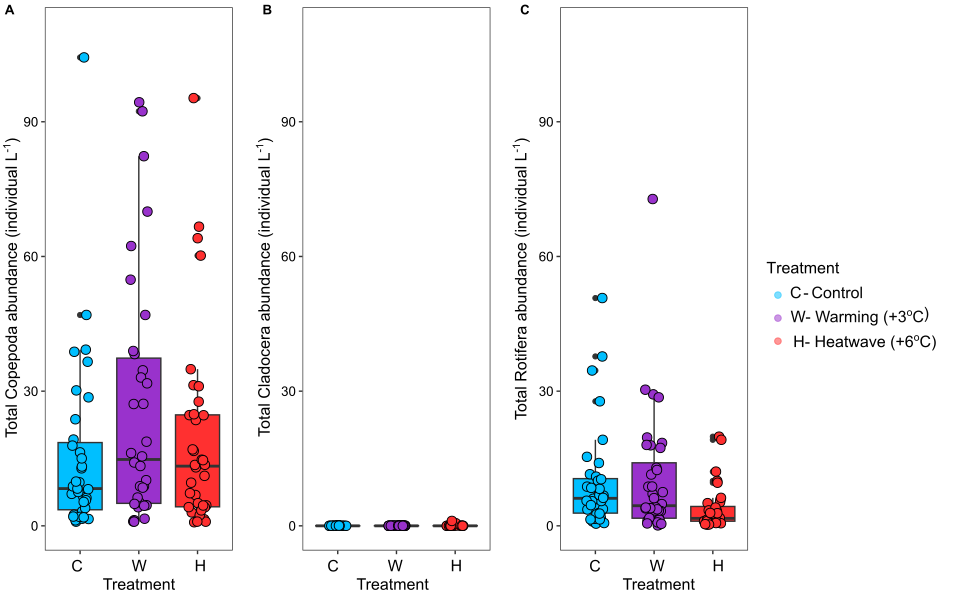


**Figure S8**. Boxplots illustrating the abundance (individual L^-1^) of **(a)** Copepoda, **(b)** Cladocera, and **(c)** Rotifera based on data collected throughout the experiment (N=36 for each treatment).


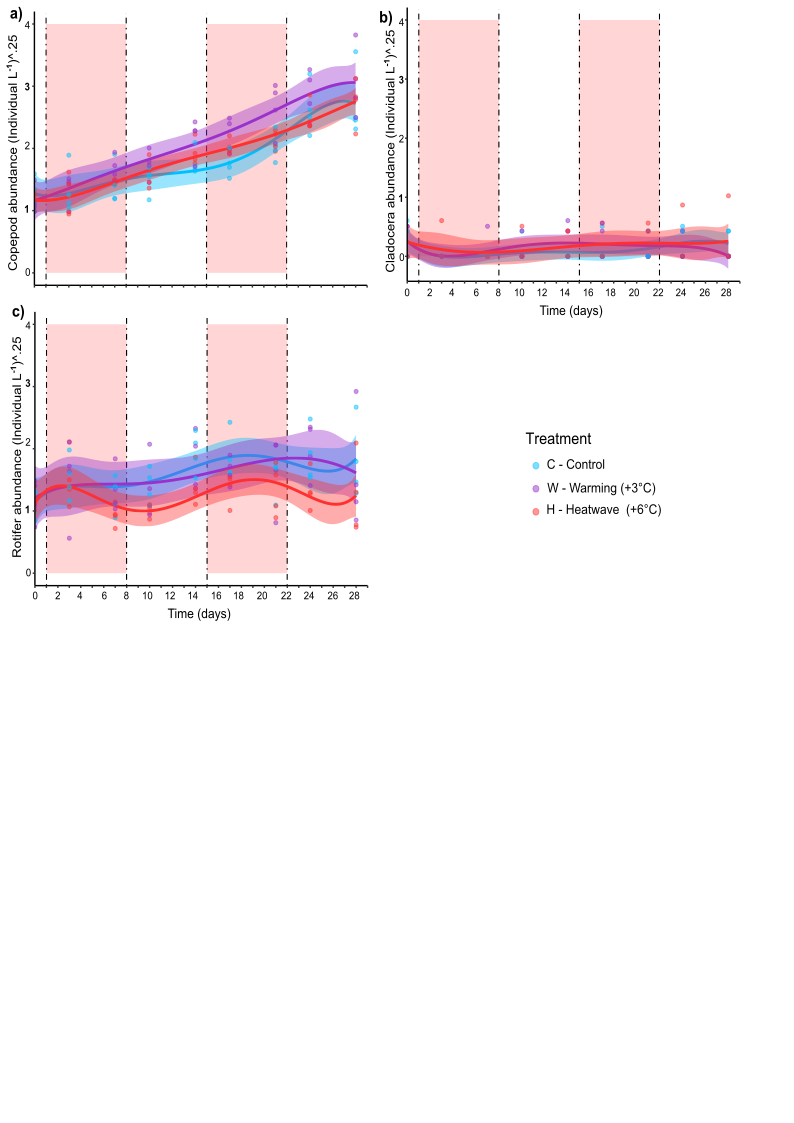


**Figure S9**. The temporal dynamics in abundance (individual L^-1^) with double square-root transformation of **(a)** Copepoda, **(b)** Cladocera, and **(c)** Rotifera during the experiment. Solid trend lines and error bands represent fitted GAMs ± SE. Red backgrounds indicate the lengths of the two experimental heatwaves.


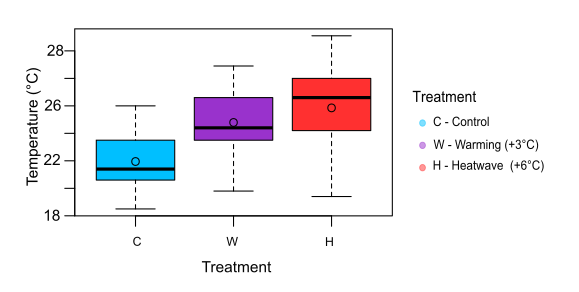


**Figure S10.** Boxplots illustrating water temperature in the different treatments based on measurements recorded during the experiment (N=16668 for each treatment). Black circles represent the mean temperatures while the middle bars represent the median temperature in each treatment.


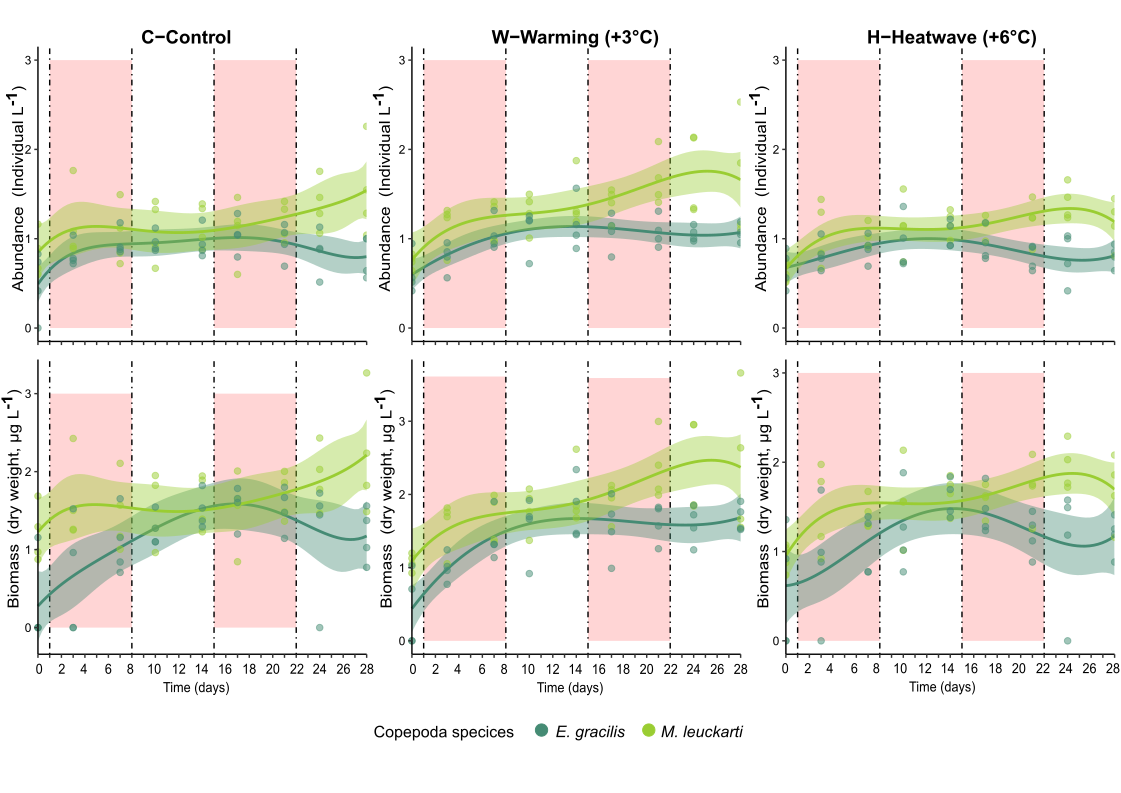


**Figure S11**. Temporal patterns in abundance (individual L^-1^) and biomass (dry weight, µg L^-1^) of the Copepoda species in the **(a)** Control**, (b)** Warming (+3°C)**,** and **(c)** Heatwave treatments (+6°C). Abundance and biomass data were double-square-root transformed for the analyses. Solid trend lines and error bands represent fitted GAMs ± SE. Red backgrounds indicate the lengths of the two experimental heatwaves.


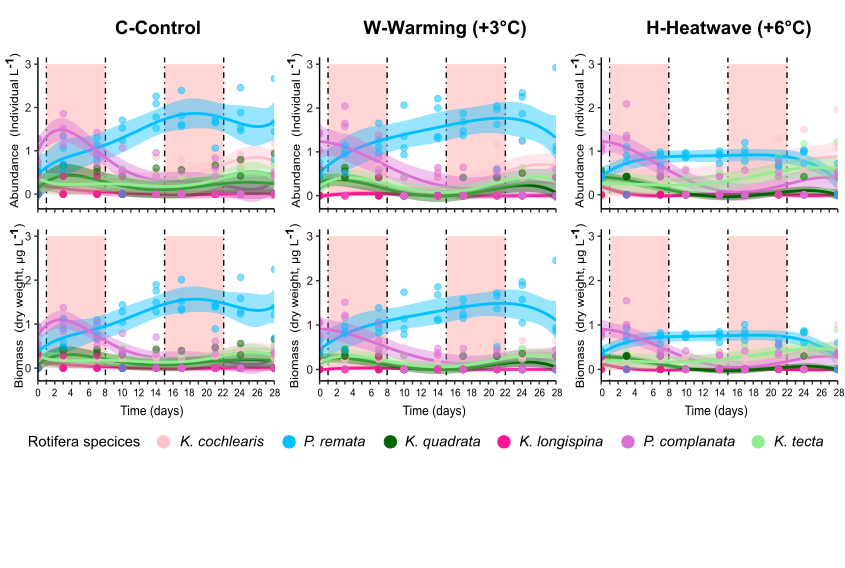


**Figure S12**. Temporal patterns in abundance (individual L^-1^ ) and biomass (dry weight, µg L^-1^) of the Rotifera species in the **(a)** Control**, (b)** Warming (+3°C)**,** and **(c)** Heatwave treatments (+6°C). Abundance and biomass data were double-square-root transformed for the analyses. Solid trend lines and error bands represent fitted GAMs ± SE. Red backgrounds indicate the lengths of the two experimental heatwaves.


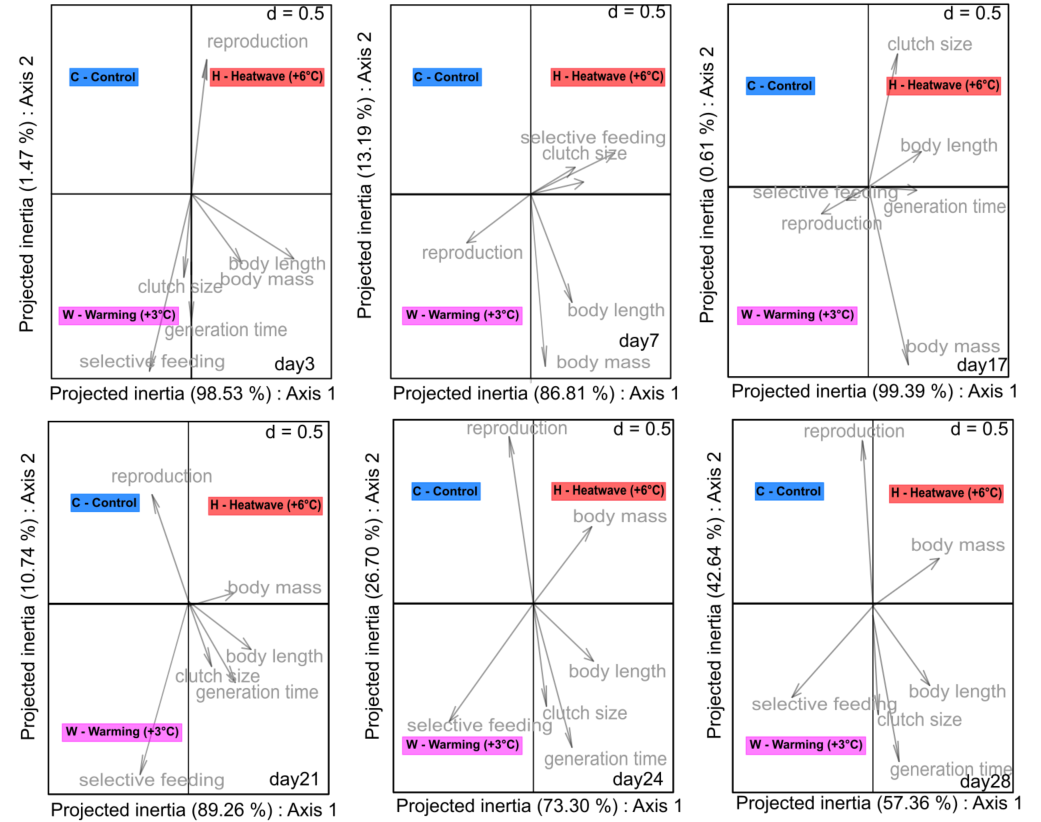


**Figure S13.** RLQ plots for days 3, 7, 17, 21, 24, and 28 show the relationships between zooplankton traits and experimental treatments. Traits that did not show a significant relationship with any of the treatments based on the fourth-corner analyses are indicated with grey arrows. (Grid size: d=0.5)


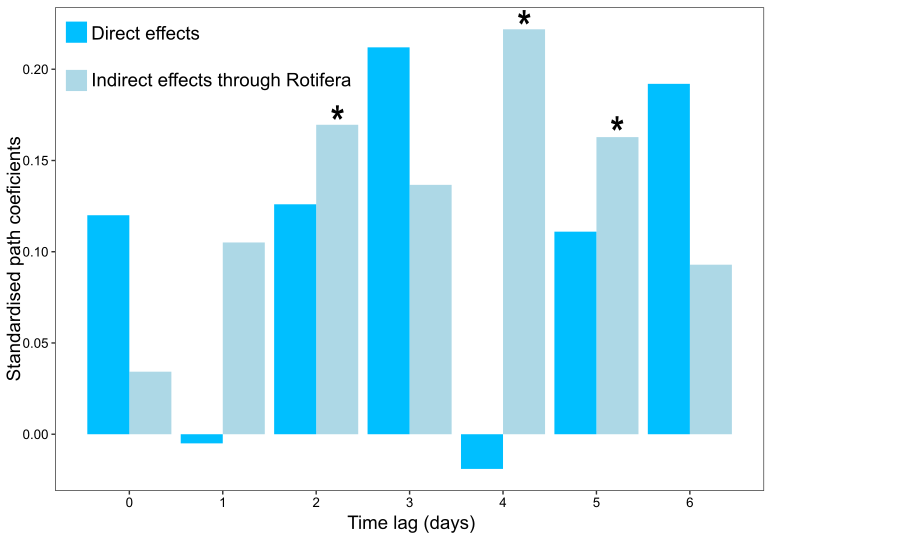


**Figure S14.** The direct and indirect effects of the heatwave treatment on phytoplankton biomass based on different time lags. Indirect effects are calculated by multiplying the path coefficient for the effect of the first experimental heatwave on Rotifera with the path coefficient for the effect of Rotifera on phytoplankton. Asterisks indicate significant effects.


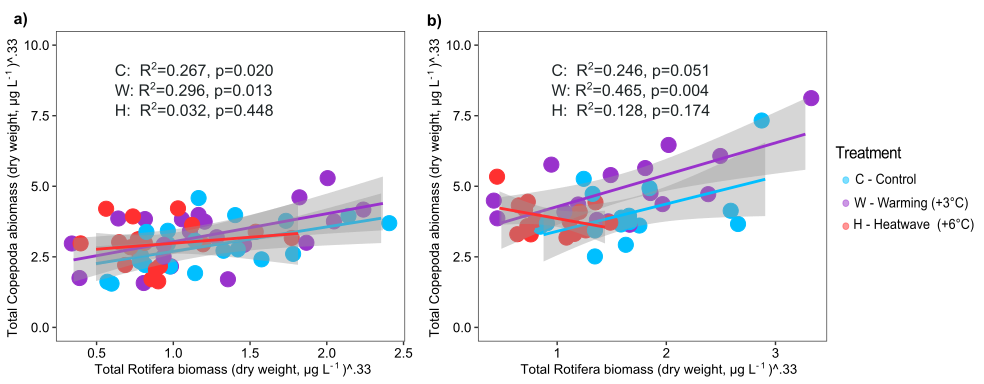


**Figure S15.** The relationships between the biomasses (dry weight, µg L^-1^, cubic-root transformed) of Rotifera and Copepoda during **(a)** the first two weeks, and **(b)** the second two weeks of the experiment within each treatment. Trend lines represent linear regression models with 95% confidence intervals.

**Table S1**. Functional traits of the zooplankton species that were included in the RLQ analysis. Average body sizes of Crustacea were obtained from the regular monitoring data of Lake Balaton, while for Rotifera, literature data were used. Body mass (expressed as dry weight) data are calculated from average body size based on length-weight regressions. Data on feeding mode were obtained from [Hlawa & Heerkloss, 1994, and Knisely & Geller, 1986](https://www.zotero.org/google-docs/?broken=AaUiOv).

| **Species** | **Body size (µm)** | **Body mass (µg)** | **Reproduction** | **Feeding mode** | **Generation time (day)** | **Clutch size** |
| --- | --- | --- | --- | --- | --- | --- |
| **Copepoda** | | | | | | |
| *Mesocyclops leuckarti* | 879.5 | 3.6 | sexual | raptorial | 15.6 [(Vijverberg, 1980)](https://www.zotero.org/google-docs/?broken=fW7Ny8) | 59.8 [(Bozkurt et al., 2016)](https://www.zotero.org/google-docs/?broken=oq4IHW) |
| *Eudiaptomus gracilis* | 1056.5 | 9.1 | sexual | raptorial | 22.7 [(Munro, 1974)](https://www.zotero.org/google-docs/?broken=RxQvWe) | 10.1 [(Poulin, 1995)](https://www.zotero.org/google-docs/?broken=Xv3nc3) |
| **Cladocera** | | | | | | |
| *Daphnia* sp. | 1246.5 | 22.8 | parthenogenic | grazer (filter feeder) | 7.5 [(McCauley et al., 1990)](https://www.zotero.org/google-docs/?broken=4zFy8J) | 3.2 [(Lynch, 1980)](https://www.zotero.org/google-docs/?broken=r9YcMY) |
| *Bosmina longirostris* | 425.0 | 1.7 | parthenogenic | grazer (filter feeder) | 6.0 [(Lynch, 1980)](https://www.zotero.org/google-docs/?broken=5ta4wG) | 2.1 [(Ślusarczyk, 1997)](https://www.zotero.org/google-docs/?broken=CbYFv2) |
| *Diaphanosoma brachyurum* | 662.0 | 4.2 | parthenogenic | grazer (filter feeder) | 6.0 [(Lynch, 1980)](https://www.zotero.org/google-docs/?broken=r9YcMY) | 6.9 [(Lemke & Benke, 2003)](https://www.zotero.org/google-docs/?broken=RpR3fM) |
| **Rotifera** | | | | | | |
| *Keratella tecta* | 109.0 [(Roche, 1993)](https://www.zotero.org/google-docs/?broken=Dh2HkZ) | 0.3 | parthenogenic | grazer (filter feeder) | 4.3 [(Walz, 1983)](https://www.zotero.org/google-docs/?broken=Zl9gqy) | 1.0 [(Bozkurt et al., 2016)](https://www.zotero.org/google-docs/?broken=zrK2xM) |
| *Keratella cochlearis* | 165.8 [(Cieplinski et al., 2018)](https://www.zotero.org/google-docs/?broken=oMK5yN) | 0.3 | parthenogenic | grazer (filter feeder) | 4.3 [(Walz, 1983)](https://www.zotero.org/google-docs/?broken=bUzeGx) | 1.0 [(Bozkurt et al., 2016)](https://www.zotero.org/google-docs/?broken=94KVkF) |
| *Keratella quadrata* | 109.0 [(Roche, 1993)](https://www.zotero.org/google-docs/?broken=jsuYv6) | 0.2 | parthenogenic | grazer (filter feeder) | 3.0 [(Pourriot & Deluzarches, 1971)](https://www.zotero.org/google-docs/?broken=E76CYf) | 1.0 [(Bozkurt et al., 2016)](https://www.zotero.org/google-docs/?broken=7e0ta4) |
| *Kellicottia longispina* | 109.0 [(Roche, 1993)](https://www.zotero.org/google-docs/?broken=ekNk56) | 0.2 | parthenogenic | grazer (filter feeder) | 5.0 [(Winberg, 1971)](https://www.zotero.org/google-docs/?broken=UKZMle) | 1.0 [(Bozkurt et al., 2016)](https://www.zotero.org/google-docs/?broken=70FVH5) |
| *Polyarthra remata* | 85.0 [(Skorikov, 1986)](https://www.zotero.org/google-docs/?HDF5dK) | 0.2 | parthenogenic | raptorial | 1.9 [(Virro, 2001)](https://www.zotero.org/google-docs/?broken=GZ86Fq) | 2.6 [(Bozkurt et al., 2016)](https://www.zotero.org/google-docs/?broken=ETyFAq) |
| *Pompholyx complanata* | 114.0 [(Gosse, 1851)](https://www.zotero.org/google-docs/?8Lfrjj) | 0.3 | parthenogenic | grazer (filter feeder) | 3.7 [(Virro, 2001)](https://www.zotero.org/google-docs/?broken=wsW9X0) | 22.6 [(Bozkurt et al., 2016)](https://www.zotero.org/google-docs/?broken=tSTwZn) |

**References**

[Bozkurt, A., Ülgü, M., & Duysak, Ö. (2016). Tahtaköprü Baraj Gölü’nde (Gaziantep, Türkiye) Zooplanktonun (Rotifera, Kladosera ve Kopepoda) Vücut Büyüklüğü ve Yumurta Miktarının Belirlenmesi. *Journal of Limnology and Freshwater Fisheries Research*, *2*(1), 1–1.](https://www.zotero.org/google-docs/?broken=iGq04v) <https://doi.org/10.17216/LimnoFish-5000169228>

[Cieplinski, A., Obertegger, U., & Weisse, T. (2018). Life history traits and demographic parameters in the Keratella cochlearis (Rotifera, Monogononta) species complex. *Hydrobiologia*, *811*.](https://www.zotero.org/google-docs/?broken=KEjHxX) <https://doi.org/10.1007/s10750-017-3499-2>

[Gosse. (1851). *Pompholyx complanata Gosse, 1851*. https://www.gbif.org/species/1000700](https://www.zotero.org/google-docs/?broken=mDeN1a)

[Hlawa, S., & Heerkloss, R. (1994). Experimental studies into the feeding biology of rotifers in brackish water. *Journal of Plankton Research*, *16*(8), 1021–1038.](https://www.zotero.org/google-docs/?broken=4kP4yi) <https://doi.org/10.1093/plankt/16.8.1021>

[Knisely, K., & Geller, W. (1986). Selective feeding of four zooplankton species on natural lake phytoplankton. *Oecologia*, *69*(1), 86–94.](https://www.zotero.org/google-docs/?broken=flXlb8) <https://doi.org/10.1007/BF00399042>

[Lemke, A., & Benke, A. (2003). Growth and reproduction of three cladoceran species from a small wetland in the south-eastern USA. *Freshwater Biology*, *48*, 589–603.](https://www.zotero.org/google-docs/?broken=z2T3gs) <https://doi.org/10.1046/j.1365-2427.2003.01034.x>

[Lynch, M. (1980). The Evolution of Cladoceran Life Histories. *The Quarterly Review of Biology*, *55*(1), 23–42.](https://www.zotero.org/google-docs/?broken=jqgHbN)

[McCauley, E., Murdoch, W. W., & Nisbet, R. M. (1990). Growth, Reproduction, and Mortality of Daphnia pulex Leydig: Life at Low Food. *Functional Ecology*, *4*(4), 505–514.](https://www.zotero.org/google-docs/?broken=tYMRdO) <https://doi.org/10.2307/2389318>

[Munro, I. G. (1974). The effect of temperature on the development of egg, naupliar and copepodite stages of two species of copepods, Cyclops vicinus uljanin and Eudiaptomus gracilis sars. *Oecologia*, *16*(4), 355–367.](https://www.zotero.org/google-docs/?broken=B1AhPO) <https://doi.org/10.1007/BF00344742>

[Poulin, R. (1995). Clutch size and egg size in free-living and parasitic Copepods: A comparative analysis. *Evolution; International Journal of Organic Evolution*, *49*(2), 325–336.](https://www.zotero.org/google-docs/?broken=73fWLr) <https://doi.org/10.1111/j.1558-5646.1995.tb02245.x>

[Pourriot, R., & Deluzarches, M. (1971). Recherches sur la biologie des rotifères. II - Influence de la température sur la durée du développement embryonnaire et post-embryonnaire. *Annales De Limnologie*, *7*(1).](https://www.zotero.org/google-docs/?broken=GTX3uB) <https://doi.org/10.1051/limn/1971008>

[Roche, K. F. (1993). Temporal variation in the morphology of the rotifer *Keratella quadrata* (Müller, 1786). *Annales de Limnologie - International Journal of Limnology*, *29*(2), 119–127.](https://www.zotero.org/google-docs/?broken=L8epMg) <https://doi.org/10.1051/limn/1993011>

[Ślusarczyk, M. (1997). Impact of fish predation on a small-bodied cladoceran: Limitation or stimulation? *Hydrobiologia*, *342*(0), 215–221.](https://www.zotero.org/google-docs/?broken=6xawzP) <https://doi.org/10.1023/A:1017083206647>

[Skorikov. (1986). *Polyarthra remata Skorikov 1896—Encyclopedia of Life*. https://eol.org/pages/1063521](https://www.zotero.org/google-docs/?broken=PdUbO7)

[Vijverberg, J. (1980). Effect of temperature in laboratory studies on development and growth of Cladocera and Copepoda from Tjeukemeer, The Netherlands. *Freshwater Biology*, *10*(4), 317–340.](https://www.zotero.org/google-docs/?broken=QW2gAn) <https://doi.org/10.1111/j.1365-2427.1980.tb01206.x>

[Virro, T. (2001). Life cycle patterns of rotifers in Lake Peipsi. *Hydrobiologia*, *446–447*, 85–93.](https://www.zotero.org/google-docs/?broken=ZTbwNp) <https://doi.org/10.1023/A:1017529323653>

[Walz, N. (1983). Individual culture and experimental population dynamics of Keratella cochlearis (Rotatoria). *Hydrobiologia*, *107*(1), 35–45.](https://www.zotero.org/google-docs/?broken=Trc6X9) <https://doi.org/10.1007/BF00126701>

[Winberg, G. G. (1971). *Methods for the estimation of production of aquatic animals*. Academic Press.](https://www.zotero.org/google-docs/?broken=Qbe2a1)

**Table S2.** Treatment-specific differences in the biomasses (based on dry weight) of total Copepoda, Cladocera, and Rotifera, total zooplankton biomass, as well as in the biomass ratio of Rotifera:Copepoda (R:C), and the biomass ratio of zooplankton:phytoplankton (ZP:PP). Summary statistics of Kruskal–Wallis tests (to test for significant treatment effects) and Dunn's tests (to test pairwise differences) are presented. Significant results (p<0.05) are indicated by bold letters, marginally significant (p<0.1) with italics, while ‘ns’ stands for non-significant (p>0.1) ones.

|  | **Date** | **Kruskal - Wallis test** | | **Dunn’s multiple comparisons tests** | | |
| --- | --- | --- | --- | --- | --- | --- |
|  |  | **Chi-squared** | **p** | **H-W** | **H-C** | **W-C** |
|  |  |  |  | **p** | **p** | **p** |
| **Copepoda biomass** | day 0 | 0.15 | ns | ns | ns | ns |
|  | day 3 | 0.03 | ns | ns | ns | ns |
|  | day 7 | 2.00 | ns | ns | ns | ns |
|  | day 10 | 1.19 | ns | ns | ns | ns |
|  | day 14 | 4.27 | ns | ns | ns | **0.04** |
|  | day 17 | *5.81* | *0.05* | *0.06* | ns | **0.02** |
|  | day 21 | **7.65** | **0.02** | **<0.01** | ns | ns |
|  | day 24 | 3.96 | ns | *0.05* | ns | ns |
|  | day 28 | 1.08 | ns | ns | ns | ns |
| **Cladocera biomass** | day 0 | 0.03 | ns | ns | ns | ns |
|  | day 3 | 1.11 | ns | ns | ns | ns |
|  | day 7 | 2.62 | ns | ns | ns | ns |
|  | day 10 | 0.05 | ns | ns | ns | ns |
|  | day 14 | 2.07 | ns | ns | ns | ns |
|  | day 17 | 0.18 | ns | ns | ns | ns |
|  | day 21 | 0.66 | ns | ns | ns | ns |
|  | day 24 | 0.25 | ns | ns | ns | ns |
|  | day 28 | 2.07 | ns | ns | ns | ns |
| **Rotifera biomass** | day 0 | 0.27 | ns | ns | ns | ns |
|  | day 3 | 0.5 | ns | ns | ns | ns |
|  | day 7 | 4.31 | ns | *0.05* | ns | ns |
|  | day 10 | 4.19 | ns | ns | *0.06* | ns |
|  | day 14 | 5.12 | *0.07* | ns | **0.03** | ns |
|  | day 17 | **7.73** | **0.02** | **0.04** | **<0.01** | ns |
|  | day 21 | 2.88 | ns | ns | ns | ns |
|  | day 24 | **6.50** | **0.03** | **0.03** | **0.02** | ns |
|  | day 28 | 3.12 | ns | ns | 0.08 | ns |
| **Zooplankton biomass** | day 0 | 0.04 | ns | ns | ns | ns |
|  | day 3 | 0.15 | ns | ns | ns | ns |
|  | day 7 | 1.88 | ns | ns | ns | ns |
|  | day 10 | 0.73 | ns | ns | ns | ns |
|  | day 14 | 3.04 | ns | ns | ns | *0.09* |
|  | day 17 | 2.88 | ns | ns | ns | ns |
|  | day 21 | **7.65** | **0.02** | **<0.01** | ns | *0.09* |
|  | day 24 | 3.85 | ns | *0.05* | ns | ns |
|  | day 28 | 0.96 | ns | ns | ns | ns |
| **Rotifera : Copepoda biomass ratio** | day 0 | 0.73 | ns | ns | ns | ns |
|  | day 3 | 0.27 | ns | ns | ns | ns |
|  | day 7 | 4.19 | ns | ns | *0.06* | ns |
|  | day 10 | 2.19 | ns | ns | ns | ns |
|  | day 14 | **7.04** | **0.03** | ns | **<0.01** | ns |
|  | day 17 | **8.35** | **0.02** | ns | **<0.01** | *0.06* |
|  | day 21 | 0.81 | ns | ns | ns | ns |
|  | day 24 | 4.50 | ns | ns | **0.03** | ns |
|  | day 28 | 1.65 | ns | ns | ns | ns |
| **Zooplankton : phytoplankton biomass ratio** | day 0 | 0.73 | ns | ns | ns | ns |
|  | day 3 | 0.04 | ns | ns | ns | ns |
|  | day 7 | 1.08 | ns | ns | ns | ns |
|  | day 10 | 2.00 | ns | ns | ns | ns |
|  | day 14 | 1.33 | ns | ns | ns | ns |
|  | day 17 | 0.81 | ns | ns | ns | ns |
|  | day 21 | 4.77 | *0.09* | **0.03** | ns | ns |
|  | day 24 | 2.92 | ns | ns | ns | ns |
|  | day 28 | 0.73 | ns | ns | ns | ns |
| **TP** | day 0 | 0.50 | ns | ns | ns | ns |
|  | day 3 | 2.42 | ns | ns | ns | ns |
|  | day 7 | 1.65 | ns | ns | ns | ns |
|  | day 10 | 0.15 | ns | ns | ns | ns |
|  | day 14 | 5.35 | *0.07* | ns | *0.07* | ns |
|  | day 17 | 2.35 | ns | ns | ns | ns |
|  | day 21 | 1.50 | ns | ns | ns | ns |
|  | day 24 | 0.73 | ns | ns | ns | ns |
|  | day 28 | 0.96 | ns | ns | ns | ns |
| **TN** | day 0 | 0.49 | ns | ns | ns | ns |
|  | day 3 | 1.61 | ns | ns | ns | ns |
|  | day 7 | 1.44 | ns | ns | ns | ns |
|  | day 10 | 0.66 | ns | ns | ns | ns |
|  | day 14 | 2.26 | ns | ns | ns | ns |
|  | day 17 | 1.46 | ns | ns | ns | ns |
|  | day 21 | 1.38 | ns | ns | ns | ns |
|  | day 24 | 1.41 | ns | ns | ns | ns |
|  | day 28 | 0.09 | ns | ns | ns | ns |
| **SRP** | day 0 | 2.85 | ns | ns | ns | ns |
|  | day 3 | 1.04 | ns | ns | ns | ns |
|  | day 7 | 1.23 | ns | ns | ns | ns |
|  | day 10 | 2.00 | ns | ns | ns | ns |
|  | day 14 | 0.05 | ns | ns | ns | ns |
|  | day 17 | 6.10 | **0.05** | ns | ns | **0.05** |
|  | day 21 | 0.48 | ns | ns | ns | ns |
|  | day 24 | 4.40 | ns | ns | ns | ns |
|  | day 28 | 1.54 | ns | ns | ns | ns |
| **NH_4_^+^** | day 0 | 0.07 | ns | ns | ns | ns |
|  | day 3 | 1.18 | ns | ns | ns | ns |
|  | day 7 | 4.06 | ns | ns | ns | ns |
|  | day 10 | 0.07 | ns | ns | ns | ns |
|  | day 14 | 0.03 | ns | ns | ns | ns |
|  | day 17 | 0.01 | ns | ns | ns | ns |
|  | day 21 | 0.40 | ns | ns | ns | ns |
|  | day 24 | 5.29 | *0.07* | ns | ns | *0.07* |
|  | day 28 | 0.21 | ns | ns | ns | ns |
| **NO_3_^-^** | day 0 | 0.88 | ns | ns | ns | ns |
|  | day 3 | 5.15 | *0.07* | *0.07* | ns | ns |
|  | day 7 | 3.20 | ns | ns | ns | ns |
|  | day 10 | 2.48 | ns | ns | ns | ns |
|  | day 14 | 2.04 | ns | ns | ns | ns |
|  | day 17 | 5.77 | *0.06* | ns | *0.08* | ns |
|  | day 21 | 3.42 | ns | ns | ns | ns |
|  | day 24 | 0.71 | ns | ns | ns | ns |
|  | day 28 | 3.85 | ns | ns | ns | ns |

**Table S3.** The effect of treatments on daily Chl*a* fluorescence over the entire experiment (excluding the first 4 days) and after splitting it into two periods resulted from a pairwise test for multiple comparisons based on the GAMM with single-step p-value adjustment.

| **Date period** | **H-W** | | | **H-C** | | | **W-C** | | |
| --- | --- | --- | --- | --- | --- | --- | --- | --- | --- |
|  | Estimate | z-value | p-value | Estimate | z-value | p-value | Estimate | z-value | p-value |
| 4-28 | 0.10 | 1.49 | ns | 0.17 | 0.47 | ns | 0.04 | 1.98 | ns |
| 4-14 | 0.24 | 3.13 | **0.005** | 0.27 | 3.53 | **0.001** | 0.03 | 0.41 | ns |
| 15-28 | 0.04 | 0.51 | ns | 0.19 | 2.60 | ns | 0.15 | 2.01 | ns |

**Table S4**. Summary of the amount of variation explained by the Structural Equation Model with different time lags during a month. Significant results (p<0.05) are indicated by bold letters, marginally significant (p<0.1) with italics, while ‘ns’ stands for non-significant (p>0.1) ones.

| **Period** | **Time lag (day)** | **AIC** | **Chl*a -* Treatment** | | **Chl*a* - Copepoda** | | **Chl*a* - Rotifera** | | **AIC** | **Rotifera - Treatment** | | **Rotifera - Copepoda** | | **Copepoda - Treatment** | | **Marginal R^2^ of the model for Chla** |
| --- | --- | --- | --- | --- | --- | --- | --- | --- | --- | --- | --- | --- | --- | --- | --- | --- |
|  |  |  | Std.Estimate | p | Std.Estimate | p | Std.Estimate | p |  | Std.Estimate | p | Std.Estimate | p | Std.Estimate | p |  |
| First 2 weeks of H | 0 | 157.798 | 0.120 | ns | *0.373* | *0.062* | -0.076 | ns | -24.901 | **-0.451** | **0.032** | 0.222 | ns | 0.057 | ns | 0.14 |
|  | 1 | 151.147 | -0.005 | ns | **0.399** | **0.047** | -0.233 | ns |  |  |  |  |  |  |  | 0.16 |
|  | 2 | 141.550 | 0.126 | ns | 0.206 | ns | **-0.376** | **0.024** |  |  |  |  |  |  |  | 0.18 |
|  | 3 | 156.157 | 0.212 | ns | -0.022 | ns | *-0.303* | *0.085* |  |  |  |  |  |  |  | 0.16 |
|  | 4 | 152.906 | -0.019 | ns | 0.122 | ns | **-0.492** | **0.007** |  |  |  |  |  |  |  | 0.19 |
|  | 5 | 142.644 | 0.111 | ns | -0.120 | ns | **-0.361** | **0.028** |  |  |  |  |  |  |  | 0.15 |
|  | 6 | 156.178 | 0.192 | ns | -0.345 | 0.063 | -0.206 | ns |  |  |  |  |  |  |  | 0.17 |
| Last 2 weeks of H | 0 | 127.658 | 0.279 | ns | 0.075 | ns | 0.019 | ns | -29.586 | **-0.560** | **0.035** | **0.279** | **0.049** | -0.266 | ns | 0.06 |
|  | 1 | 138.983 | 0.224 | ns | 0.132 | ns | -0.181 | ns |  |  |  |  |  |  |  | 0.10 |
|  | 2 | 126.023 | 0.065 | ns | 0.057 | ns | -0.249 | ns |  |  |  |  |  |  |  | 0.06 |
|  | 3 | 133.255 | 0.004 | ns | *0.258* | *0.084* | ***-0.438*** | ***0.030*** |  |  |  |  |  |  |  | 0.13 |
|  | 4 | 132.794 | 0.280 | ns | 0.008 | ns | -0.033 | ns |  |  |  |  |  |  |  | 0.07 |
|  | 5 | 129.095 | 0.535 | ns | -0.081 | ns | *0.371* | *0.053* |  |  |  |  |  |  |  | 0.15 |
|  | 6 | 125.087 | 0.276 | ns | 0.023 | ns | 0.111 | ns |  |  |  |  |  |  |  | 0.04 |
| First 2 weeks of W | 0 | 144.485 | -0.162 | ns | 0.349 | ns | 0.105 | ns | -37.116 | -0.107 | ns | 0.335 | ns | 0.290 | ns | 0.12 |
|  | 1 | 151.799 | -0.197 | ns | 0.356 | ns | -0.112 | ns |  |  |  |  |  |  |  | 0.09 |
|  | 2 | 151.612 | 0.152 | ns | -0.102 | ns | -0.049 | ns |  |  |  |  |  |  |  | 0.03 |
|  | 3 | 151.551 | 0.221 | ns | -0.267 | ns | -0.133 | ns |  |  |  |  |  |  |  | 0.10 |
|  | 4 | 155.550 | -0.065 | ns | 0.116 | ns | *-0.312* | *0.094* |  |  |  |  |  |  |  | 0.07 |
|  | 5 | 147.369 | 0.141 | ns | -0.131 | ns | -0.274 | ns |  |  |  |  |  |  |  | 0.10 |
|  | 6 | 134.663 | 0.111 | ns | -0.236 | ns | *-0.281* | *0.067* |  |  |  |  |  |  |  | 0.14 |
| Last 2 weeks of W | 0 | 154.747 | 0.157 | ns | 0.219 | ns | -0.074 | ns | -12.679 | -0.302 | ns | **0.670** | **<0.001** | 0.358 | ns | 0.07 |
|  | 1 | 160.011 | 0.178 | ns | 0.221 | ns | *-0.281* | *0.085* |  |  |  |  |  |  |  | 0.09 |
|  | 2 | 148.274 | 0.144 | ns | 0.131 | ns | *-0.287* | *0.071* |  |  |  |  |  |  |  | 0.07 |
|  | 3 | 151.511 | 0.117 | ns | **0.445** | **0.011** | **-0.538** | **0.003** |  |  |  |  |  |  |  | 0.18 |
|  | 4 | 153.659 | 0.210 | ns | 0.239 | ns | -0.285 | ns |  |  |  |  |  |  |  | 0.11 |
|  | 5 | 152.473 | 0.438 | ns | -0.165 | ns | 0.229 | ns |  |  |  |  |  |  |  | 0.15 |
|  | 6 | 155.321 | 0.279 | ns | 0.035 | ns | -0.032 | ns |  |  |  |  |  |  |  | 0.07 |
